# Detecting the limits of regulatory element conservation and divergence estimation using pairwise and multiple alignments

**DOI:** 10.1186/1471-2105-7-376

**Published:** 2006-08-14

**Authors:** Daniel A Pollard, Alan M Moses, Venky N Iyer, Michael B Eisen

**Affiliations:** 1 Graduate Group in Biophysics, University of California, Berkeley, CA 94720, USA; 2 Department of Molecular and Cell Biology, University of California, Berkeley, CA 94720, USA; 3 Department of Genome Sciences, Genomics Division, Ernest Orlando Lawrence Berkeley National Lab, Berkeley, CA 94720, USA; 4 Center for Integrative Genomics, University of California, Berkeley, CA 94720, USA

## Abstract

**Background:**

Molecular evolutionary studies of noncoding sequences rely on multiple alignments. Yet how multiple alignment accuracy varies across sequence types, tree topologies, divergences and tools, and further how this variation impacts specific inferences, remains unclear.

**Results:**

Here we develop a molecular evolution simulation platform, CisEvolver, with models of background noncoding and transcription factor binding site evolution, and use simulated alignments to systematically examine multiple alignment accuracy and its impact on two key molecular evolutionary inferences: transcription factor binding site conservation and divergence estimation. We find that the accuracy of multiple alignments is determined almost exclusively by the pairwise divergence distance of the two most diverged species and that additional species have a negligible influence on alignment accuracy. Conserved transcription factor binding sites align better than surrounding noncoding DNA yet are often found to be misaligned at relatively short divergence distances, such that studies of binding site gain and loss could easily be confounded by alignment error. Divergence estimates from multiple alignments tend to be overestimated at short divergence distances but reach a tool specific divergence at which they cease to increase, leading to underestimation at long divergences. Our most striking finding was that overall alignment accuracy, binding site alignment accuracy and divergence estimation accuracy vary greatly across branches in a tree and are most accurate for terminal branches connecting sister taxa and least accurate for internal branches connecting sub-alignments.

**Conclusion:**

Our results suggest that variation in alignment accuracy can lead to errors in molecular evolutionary inferences that could be construed as biological variation. These findings have implications for which species to choose for analyses, what kind of errors would be expected for a given set of species and how multiple alignment tools and phylogenetic inference methods might be improved to minimize or control for alignment errors.

## Background

Annotation of *cis*-regulatory sequences, non-coding RNAs and other functional noncoding sequences is a major challenge in molecular genetics today. Whole genome sequences of closely related species, such as those now available in mammals, flies, worms, yeast and bacteria, provide an opportunity for evolutionary analyses to greatly aid in this effort, but also present new challenges for sequence analysis [1].

The first step in studying the evolution of noncoding sequences is alignment. New tools have been developed for fast and accurate alignment of long stretches of genomic sequence (reviewed in [2-4]) and benchmarking studies have begun to address the accuracy of these pairwise [5,6] and multiple [7,8] alignment tools under various evolutionary scenarios. Knowing the nucleotide-level accuracy of alignment tools greatly informs decisions about which tools to use and which species to compare, but the impact of alignment error on evolutionary studies of noncoding sequences is only just beginning to be explored [6,8].

Sophisticated molecular evolution models and tests have been developed over the last few decades to identify various forms of selection and sequence features, yet their application nearly always assumes a perfect alignment [9]. It is commonly appreciated that highly diverged species align poorly and therefore are unsuitable for many alignment based evolutionary inferences. Thus cautious researchers tend to study recently diverged species that align trivially, but which have the potential to not be as informative as more diverged species. Ideally one would use the set of species that maximize information for an acceptable amount of error in an estimate.

Because of the inferential nature of evolutionary studies, no experiment in extant taxa could generate information about the true orthology of sequences, so simulations offer a tractable alternative. Molecular evolution simulations have been used to assess evolutionary analysis methods, including divergence estimation [10,11] and phylogeny reconstruction methods [12-15], as well as protein [16,17] and non-coding alignment accuracy [5-8,18,19].

Here we present the results from a simulation-based study assessing the accuracy of multiple alignments and the effect of alignment accuracy on two fundamental evolutionary inferences: transcription factor binding site conservation and divergence distance estimation.

The most frequent noncoding targets of comparative analyses are *cis*-regulatory DNAs that contain functional binding sites for transcription factors and thereby control gene expression [20]. Although transcription-factor binding sites are generally more conserved than surrounding sequences [21-34], they have also been observed to be gained and lost through evolution [35-42]. Precise measurements of binding site conservation, therefore, are essential for studying their evolutionary dynamics as well as identifying regulatory regions.

Divergence estimates inform nearly all evolutionary analyses. Accurate measurements of noncoding divergences are used for many purposes including differentiating functional from non-functional sequences based on constraint [43-51], showing lineage specific rate changes [52,53] and as a baseline for comparing other kinds of rates, like binding site gain and loss [38].

Below we first examine multiple alignment accuracy across tools, sequence types, trees and divergences. We show that multiple alignment accuracy is primarily determined by the pairwise divergence of the two most diverged species. We next look at alignment accuracy of transcription factor binding sites. We show that although they align better than their surrounding noncoding DNA, they are misaligned at a high enough frequency such that precise studies of gain and loss events could easily be confounded by alignment errors. Finally we look at the impact multiple alignment accuracy has on divergence distance estimation. We show that divergences tend to be overestimated at short distances and cease to increase at a tool specific maximum divergence, corresponding to the point at which alignment accuracy reaches its minimum. We also show that overall alignment accuracy, binding site alignment accuracy and divergence estimation accuracy vary across branches in a tree such that terminal branches are aligned better than internal branches. Implications for method development and evolutionary analysis are discussed.

## Results

### CisEvolver

For the purposes of this study we developed a molecular evolution simulator, CisEvolver, that incorporates several known characteristics of noncoding sequences. CisEvolver takes an ancestral DNA sequence and evolves it along a mutation guide tree, producing sequences for which we know the true alignment. The utility of such a simulation is that the sequences can be re-aligned using standard alignment tools and the accuracy of the tool alignment as well as the accuracy of any inference from the tool alignment can be measured by comparison with the true alignment. In cases where the error in an inference is due to both alignment error and error in the inference method itself, the contribution of alignment error to the total inference error can be directly measured by comparison of inference from the tool alignment and inference from the true alignment.

We implemented CisEvolver with two types of sequences, background genomic sequence and transcription factor binding sites. Background genomic sequences are evolved according to the Hasegawa Kashina Yano 1985 (HKY85) substitution model [54], a Poisson insertion/deletion (indel) event model and an empirical indel length frequency distribution [55]. Transcription factor binding sites are evolved according to the Halpern Bruno 1998 (HB98) model of position specific substitution rates [56,57], which requires the less degenerate positions in a transcription factor binding site to evolve more slowly and more specifically according to a position specific weight matrix [58] (see Methods for more details).

CisEvolver is freely available [59].

### Simulations & alignments

**Figure 1:**
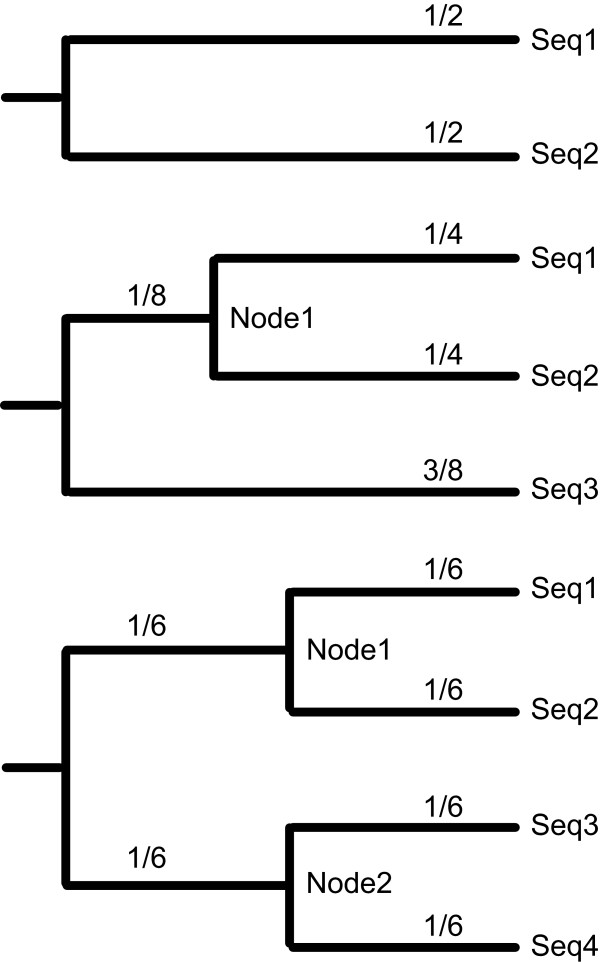
**Mutation Guide Trees**. Simulations were performed on two, three and four species trees. Numbers on the branches indicate the fraction of the total tree divergence distance on each branch.

Using CisEvolver we simulated a large set of alignments on which downstream analyses were performed. Sequences were simulated over a range of total divergence distances on two, three and four species trees with fixed topologies and fixed branch length proportions as depicted in figure 1. The relative branch lengths in these three topologies were chosen for direct comparisons of branches within the tree, as discussed below (see Alignment Accuracy). Two basic classes of sequences were simulated representing either 10 kb background genomic sequences or variable length enhancer sequences. Background genomic sequences were simulated with uniform substitution and indel rates. Enhancer sequences were evolved from 36 experimentally characterized regulatory regions from *Drosophila melanogaster *[26,60] containing the binding sites for eight transcription factors with known binding specificity: Bicoid, Caudal, Giant, Hunchback, Knirps, Kruppel, Tailless and Torso-Response Element [60-62]. Binding sites within the enhancers were evolved using CisEvolver's binding site evolution model with no gain or loss events and surrounding sequences were evolved as genomic background with substitutions and indels (see Methods for more details). One hundred replicates and 25 replicates for each divergence and tree topology were generated for background genomic sequences and each of the 36 enhancers respectively.

All alignments were performed using default parameter settings for Clustalw [63], Mavid [64], Mlagan [65] and Blastz/Tba [7,66,67] (see Methods for details). These tools were chosen based on their usage, availability, speed and ability to produce collinear multiple alignments of large genomic regions and were meant to be representative of algorithms and parameter settings. We note that Blastz/Tba is a local alignment tool and therefore, unlike the global alignment tools, does not always return an alignment. Finally, although we present the relative performance of these specific tools, our focus in this study is on the relationship of their accuracy with evolutionary scenarios and the inferences that can be made from their alignments.

### Alignment accuracy

**Figure 2:**
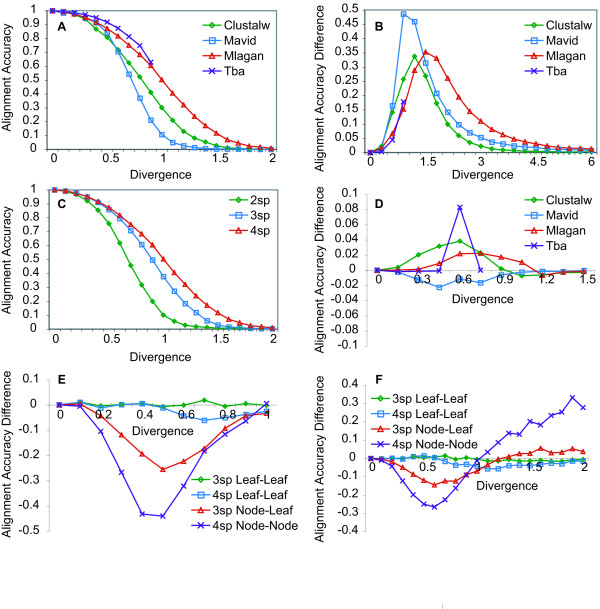
**Multiple Alignment Accuracy**. A: Alignment accuracy varies across tools and divergences. Mean four species alignment accuracy for each tool was measured as a function of total divergence distance. B: Alignment accuracy improves with the presence of transcription factor binding sites. Mean improved alignment accuracy of enhancers over background sequences for four species alignments was measured as a function of total divergence distance. C: Dividing a fixed total divergence up with more species improves alignment accuracy. Mean Mlagan alignment accuracy for two, three and four species trees was measured as a function of total divergence distance. D: Adding in-group species to a pair of species has no effect on the alignment accuracy of the pair. Mean improved alignment accuracy of three species alignments over two species alignments, where the divergence distance between Seq1 and Seq3 in the three species alignment was the same as the divergence distances between Seq1 and Seq2 in the two species alignment, was measured as a function of divergence distance. E & F: Alignment accuracy varies across branches in a tree and is best for leaf-to-leaf alignments and worst for node-to-node alignments, with the exception of highly diverged enhancers. Mean Clustalw alignment accuracy along branches in three and four species trees subtracted from mean two species alignment accuracy, where divergence along each branch is the same as the two species divergence, was measured in background sequences (E) and enhancers (F) as a function of divergence distance.

Using simulated true alignments and tool alignments we characterized the variation in alignment accuracy across alignment tools, divergences and trees. Alignment accuracy was defined as the fraction of ungapped columns in a true alignment that were aligned identically in a tool alignment (see Methods & "sensitivity" in [5]). We examined many aspects of pairwise and multiple alignment accuracy and our major observations were:

i. Alignment accuracy varies across tools and divergences (figure 2A).

ii. The presence of transcription factor binding sites leads to higher alignment accuracy (figure 2B).

iii. More species results in better accuracy when comparing trees of equal total divergence but different numbers of leaves (figure 2C).

iv. The improvement of adding a fourth species is less than that of adding a third when comparing trees of equal total divergence but different numbers of leaves (figure 2C).

v. Adding in-group species or out-group species to a pair of species has an insignificant effect on the alignment accuracy of the pair (figures 2D, 2E &2F).

In addition to these investigations into alignment accuracy across all species in alignments, we also examined the alignment accuracy for subsets of species within multiple alignments, attempting to relate the accuracy to the tree topology. We measured what we call leaf-to-leaf accuracy, node-to-leaf accuracy and node-to-node accuracy (see Methods). Leaf-to-leaf accuracy refers to the accuracy of the alignment of sister taxa (i.e. seq3 to seq4 in the four species alignments in figure 1), conditioned on the columns being ungapped across all the sequences. Node-to-leaf accuracy refers to the accuracy of the three species alignments, conditioned on the columns containing correct alignments of seq1 to seq2. Node-to-leaf accuracy therefore only depends on the alignment accuracy of node1 to seq3. Similarly, node-to-node accuracy refers to the accuracy of the four species alignments, conditioned on the columns containing correct alignments of seq1 to seq2 and seq3 to seq4. Node-to-node accuracy therefore only depends on the alignment accuracy of node1 to node2. Using these measures also found that:

vi. Leaf-to-leaf alignments are more accurate than node-to-leaf alignments, which are more accurate than node-to-node alignments, with the exception of highly diverged enhancers (figures 2E &2F).

Observations i and ii were consistent with our expectations. Although all four tools in this study use some form of the Needleman-Wunsch algorithm, they each utilize unique algorithmic features and scoring schemes, leading to variation in their alignments and therefore alignment accuracy under different evolutionary conditions (figure 2A). Both, the decrease in alignment accuracy with greater divergence distance (figure 2A) as well as the increase in alignment accuracy with the addition of transcription factor binding sites (figure 2B), are the expected outcome of higher similarity and fewer indels leading to higher alignment accuracy (as we have previously reported for pairwise alignments [5]).

Our results on the relationship of alignment accuracy to the number of species aligned (observations iii, iv and v) are consistent with the hypothesis that the pairwise distance between the two most diverged species in a tree effectively determines alignment accuracy. Across tools and divergences, adding ingroup or outgroup species to a pair of species of fixed divergence had an insignificant effect on alignment accuracy (t-test, p > 0.05) (figure 2D and leaf-to-leaf accuracy in 2E &2F). Brudno et al found Mlagan alignments of human and fugu exons were improved by 3% with the addition of mouse as an in-group [65], which is consistent with the trend we observed with Mlagan alignments improving with in-group addition, but this trend was not found to be highly significant at any divergence. Observations iii and iv, that dividing a fixed total divergence up with more species improves accuracy incrementally (figure 2C), may appear to be in conflict with this hypothesis but are in fact consistent. The increase in alignment accuracy with additional species dividing up a fixed total divergence is due to a decrease in the pairwise divergence between the two most diverged species, not the actual addition of species (figures 2D, 2E &2F). Thus the span of the two most diverged species, not the number of species in the alignment, appears to be the primary determinant of alignment accuracy.

Finally, observation vi, that alignment accuracy varies across branches in a tree, is quite unexpected. The progressive alignment steps that these four tools use appear to be biased toward aligning leaf sequences better than internal nodes, where sub-alignments must be aligned (figure 2E). This bias was found to be inconsistent for enhancer sequences, for which alignment accuracy of node-to-node and node-to-leaf branches actually were better than leaf-to-leaf branches at high divergences (figure 2F). This variation is surprising given that the accuracy of the alignment of a node to another node or sequence is conditioned on the sequences below that node (in the tree) having been aligned correctly (see Methods). These results suggest that the step of aligning sub-alignments is harder than aligning sequences, consistent with the idea that progressive alignment heuristics often lead to sub-optimal alignments [68]. Variation of alignment accuracy across branches in a tree has profound implications for phylogenetic analysis.

To understand the relationship of the observed variation in alignment accuracy with phylogenetic analyses performed using automated alignments, we explored the following two evolutionary inferences.

### Transcription factor binding site alignment

Using simulated true alignments and tool alignments of enhancers containing conserved transcription factor binding sites we examined the accuracy of binding site alignment and its relationship with overall alignment accuracy. We used two definitions of binding site alignment. Aligned sites were classified as either perfectly aligned, meaning every base in the binding site was aligned correctly across all species, or overlapping, meaning the binding sites across the species overlapped at at least one position (similar to definitions in [34]).

**Figure 3:**
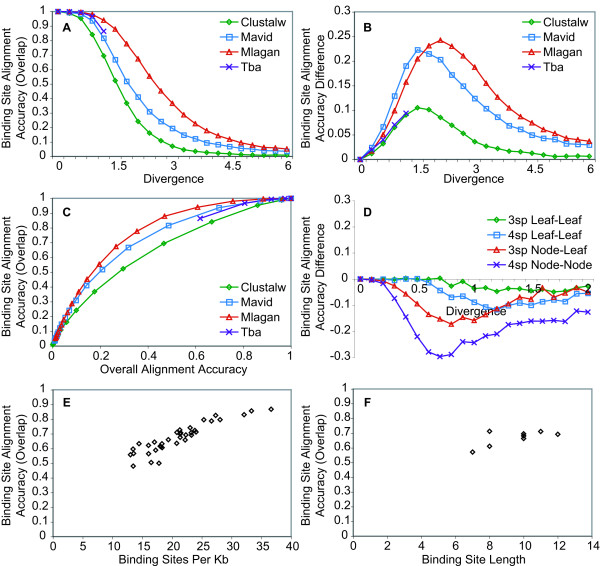
**Transcription Factor Binding Site Alignment Accuracy**. A: Binding site alignment accuracy varies across tools and divergences. Fraction of binding sites overlapping in four species alignments was measured as a function of total divergence distance. B: Binding sites are often still overlapping in alignments even when they are not perfectly aligned. Fraction of binding sites perfectly aligned in four species alignments subtracted from the fraction of binding sites overlapping in four species alignments was measured as a function of total divergence distance. C: Binding site alignment accuracy is highly correlated with overall alignment accuracy and is consistently higher. Fraction of binding sites overlapping in four species alignments was measured as a function of overall alignment accuracy. D: Binding site alignment accuracy varies across branches in a tree and is best for leaf-to-leaf alignments and worst for node-to-node alignments. Fraction of binding sites overlapping along branches in three and four species trees subtracted from the fraction of binding sites overlapping in two species Clustalw alignments, where the divergence along each branch is the same, was measured as a function of divergence distance. E: Binding site alignment accuracy is positively correlated with binding site density in an enhancer. Fraction of binding sites overlapping in replicate four species Mlagan alignments of each of the 36 enhancers was measured as a function of the density of binding sites in the enhancer. F: Binding site alignment accuracy is positively correlated with binding site length. Fraction of binding sites overlapping in four species Mlagan alignments for each of the eight transcription factors was measured as a function of the length of the transcription factors' binding sites.

We first looked to see if binding site alignment accuracy varies across tools and divergences. Indeed, across tools binding alignment accuracy is a decreasing function of divergence distance. Figure 3A shows the fraction of sites overlapping in four species enhancer alignments.

We next compared our two binding site alignment scores. We were somewhat surprised to see how different the two scores are, based on the intuition that conserved binding sites should make for good anchors and large indels in flanking sequences therefore ought to be the cause of most alignment errors. Instead it appears that binding sites are often still overlapping in an alignment even if they are not perfectly aligned. Figure 3B shows the difference between our two scores in four species alignments. The large difference between the two scores suggests that evolved binding sites might not be strong anchors and therefore alignment errors in regulatory regions may often be subtle.

We next looked to see how binding site alignment accuracy is related to overall alignment accuracy. Across tools, divergence distances and trees, binding site alignment accuracy is highly correlated with overall alignment accuracy, however, binding site alignment accuracy is consistently higher than overall alignment accuracy. Figure 3C shows overlap binding site accuracy as a function of overall alignment accuracy for four species alignments. Similar to overall alignment accuracy of enhancers (figure 2F), binding site alignment accuracy also varies across branches in trees (figure 3D).

Lastly, we looked at properties of enhancers and binding sites to see how they are related to binding site alignment accuracy. We expected that enhancers with a greater density of binding sites would align more easily. Indeed, across tools, divergence distances and trees, binding site alignment accuracy is strongly and significantly correlated with the density of binding sites in an enhancer (figure 3E, Spearman's *rho *= 0.92 p < 10^-10^). We also looked at the length and average information content of binding sites to see if longer or more highly specified sites tend to align better. Across tools, divergence distances and trees, binding site alignment accuracy is correlated with binding site length (figure 3F, Spearman's *rho *= 0.44 p < 0.3) and average information content (Spearman's *rho *= 0.40 p < 0.35) but neither correlation is significant, likely because of the small number of factors used in this study. Thus the greater the density and the longer and more specified the sites in an enhancer, the more likely the sites will be aligned correctly.

### Divergence estimation

Using simulated true alignments and tool alignments of 10 kb background noncoding sequences we investigated the effects of alignment errors on divergence estimation. Divergence distances were estimated from alignments using the Baseml program from the PAML package [69] (see Methods for run parameters). We used divergence estimation error, instead of accuracy, so as to capture the directionality of errors (overestimated or underestimated). We defined it as the fractional difference between the Baseml estimate and the true divergence used in the simulation: (Estimate – True)/True.

**Figure 4:**
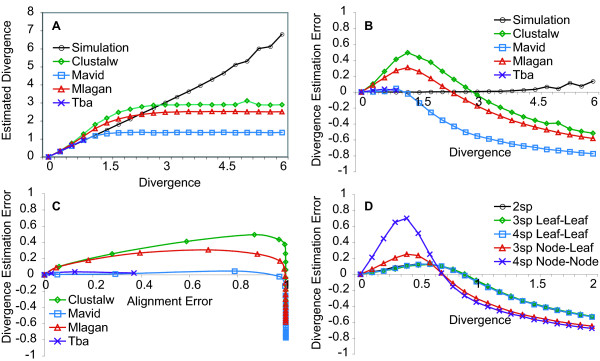
**Divergence Distance Estimation**. Divergences estimated from tool alignments are overestimated at short divergence distances and underestimated at large divergence distances while divergences estimated from true simulated alignments are accurate to large divergence distances. A: Mean divergence distance estimated from simulated alignments and tool alignments for four species trees was measured as a function of total true divergence distance. B: Mean divergence estimation error (Estimate – True/True) for four species trees was measured as a function of total true divergence distance. C: Divergence estimation error from tool alignments is not correlated with alignment error. Mean divergence estimation error for four species trees was measured as a function of mean alignment error. D: Divergence estimation error varies across branches in a tree and is best for leaf-to-leaf alignments and worst for node-to-node alignments. Mean divergence estimation error along branches of equal true divergence from two, three and four species Mlagan alignments was measured as a function of true divergence distance.

We first checked to see if divergence estimates from the simulated alignments are accurate. Indeed out to high divergence distances, Baseml estimates are very close to input divergences (figure 4).

We next looked to see if and how divergence estimation accuracy varies across tools and divergences. Our expectation was that divergence estimation accuracy would steadily decrease with divergence distance at a tool specific rate, as alignment accuracy does. Instead we found estimated divergences tend to be mostly accurate (or somewhat overestimated) at short divergence distances but are always underestimated at long divergence distances. Figure 4A shows divergence estimates from four species alignments across tools and divergences. Figure 4B shows the same data presented as divergence estimation error, as a function of true divergence distance. Perhaps most striking is the asymptotic approach of estimates to tool specific maxima. This result is consistent with Shabalina and Kondrashov's findings that the alignment of random sequences results in a percent identity much greater than the random expectation of the sum of the squared base frequencies [70]. If diverging sequences evolve to a lower identity than that of random sequences then alignment tools treat them like they are random and produce an alignment that has a fixed divergence. Indeed aligned random sequences produce similar divergences as the observed maximum divergences from our simulations (data not shown). Interestingly, the two tools that have the highest maximum divergence (Clustalw and Mlagan) both overestimate divergences at short divergence distances while the two other tools do not. Finally, Tba, the only local alignment tool in our analysis, stops returning alignments before it reaches its maximum divergence, indicating that the algorithm can avoid aligning random alignments but therefore also cannot return weakly informative alignments at large divergence distances.

Because divergence estimation accuracy appears to vary in different ways than alignment accuracy, we looked directly at their relationship. Figure 4C shows four species divergence estimation error as a function of alignment error. With the exception of Tba, which stops returning alignments while alignment error is still small, tools reach the point at which divergence estimates cease to increase close to the point at which alignment accuracy reaches its minimum. The accuracy of divergence estimates from Mavid may be due to the fact that it requires a tree with branch lengths and we provided the true divergences. The accuracy of divergence estimates from the other three tools is remarkable given the poor quality of the alignments at long divergence distances.

We last looked to see if divergence estimation accuracy varies across branches in trees as alignment accuracy does. Across tools, divergence estimates were most accurate for leaf-to-leaf branches, less accurate for node-to-leaf branches and least accurate for node-to-node branches. Figure 4D shows the error in divergence estimates from Mlagan alignments of leaf-to-leaf, node-to-leaf and node-to-node branches in two, three and four species trees. Mlagan's tendency to overestimate divergence distances at short divergence distances and to underestimate divergence distances at long divergence distances is least pronounced in leaf-to-leaf alignments and most pronounced in node-to-node alignments. The point at which divergence distances cease to increase also appears to be at a shorter divergence distance for node-to-node branches than leaf-to-leaf branches, reflecting the lower alignment accuracy of those branches. The variation in divergence estimation accuracy across branches in a tree has significant implications for phylogenetic analysis of DNA sequences.

## Discussion

Molecular evolutionary studies of noncoding DNA have either relied on the intuition that closely related species can be aligned well or have ignored alignment error all together [1-4,9]. To gain perspective on how alignment might impact evolutionary analysis, we investigated multiple alignment accuracy and its relationship with two fundamental evolutionary inferences: transcription factor binding site conservation and divergence estimation.

Because gold standards for base-level noncoding and regulatory DNA alignment accuracy do not exist, we developed a simulation platform called CisEvolver that can evolve background noncoding DNA, transcription factor binding site DNA or a mixture of the two (enhancers). We implemented CisEvolver with features of background and regulatory sequence evolution that are well modeled and are present in most comparative systems. Certainly more complicated evolutionary phenomena have been observed, and in cases where modeling is successful, ought be the subject of future studies. For instance, substitution rates have been shown to vary across sequences and have been modeled in various ways, most commonly using a gamma distribution [71]. In our study we modeled both substitution and indel rate variation using interspersed transcription factor binding sites, but rates may vary for additional reasons other than regulatory constraints, in which case a gamma distribution in our background model may be appropriate. Interestingly, a recent study showed that using a gamma distribution in simulations has no effect on Clustalw alignment accuracy when comparing sequences with the same overall identity [6], suggesting that our results are likely robust to rate variation. Compensatory substitutions (like those observed in structural noncoding RNAs) [72-74], ancient and lineage specific repetitive sequences (like those common in mammals), inversions and rearrangements [75,76] could all be incorporated into the CisEvolver platform for alignment analysis. As models of the *cis*-regulatory code [77] and binding site evolution [38,57] are developed, they too should be tested for affects on alignment accuracy. Additionally, the trees we chose to study are idealistic, in that they are ultrametric (leaves are equidistant from parent nodes), and they contain relatively few species compared to many real datasets. Trees with rate changes across many lineages would likely present additional problems that should be examined in future studies. A comprehensive analysis of the influence of tree shapes on alignment would be an interesting future direction (see [8] for an initial analysis). Despite the absence of these more complicated or unexplored aspects of noncoding evolution in the current study, our results suggest that even under these simple and ideal circumstances numerous issues arise from alignment error that ought to be qualitatively informative for all systems.

Using alignments generated by CisEvolver we tested the accuracy of alignments generated by four commonly used genomic alignment tools. All tools were run using their default parameter values (see Methods). It is quite possible that the accuracy of the alignments generated by some of these tools is highly sensitive to parameter settings and scoring schemes. In this study we focused on consistent behavior across tools and also how variation influenced inferences and were therefore not concerned with the relative performance of each tool. In order for users to optimize the use of current tools and also in order for designers of alignment tools to understand which algorithmic innovations actually improve alignment accuracy (beyond parameter settings), a systematic analysis of parameters is needed. In this study, using just default parameters, we found that the primary determinant of multiple alignment accuracy is the pairwise divergence distance between the two most diverged species in the alignment (figure 2D). Although dividing up a given divergence distance by more species improves accuracy (figure 2C), this appears to be simply due to the decrease in pairwise divergence separating the two most diverged species. Although we found that adding additional species (either in-groups or out-groups) to two species of a fixed divergence distance had an insignificant and inconsistent (across tools) impact on alignment accuracy (figure 2D), a concurrent study found that Clustalw alignments are most improved when an additional species is added at a distance equal to one third the pairwise distance separating two other species [8] (which we note is the topology we used in this study; see figure 1). Brudno et al also found that adding mouse to human-fish alignments improved Mlagan alignments by 3% [65]. If there is an affect of adding an in-group, our results suggest that it is weak and is not robust to alignment tool choice. Perhaps our most striking finding is that the accuracy of alignments varies across branches in a tree such that they are most accurate for alignments of sister taxa and least accurate between internal nodes that align sub-alignments. As we discuss below, this variation in accuracy causes variation in inferences across the tree, which could easily be construed as lineage specific biological variation. Future development of tools that minimize this distortion in accuracy across branches in a tree will be extremely valuable.

The first evolutionary inference we examined was the measurement of the conservation of transcription factor binding sites in regulatory regions. Studies have used conservation of binding sites as either a means of classifying functional from spurious predictions [21-33] or for the purposes of understanding their rates of change, or turnover [35-42]. Here we wanted to understand how far out such estimates might be accurate, what approaches might be taken to improve such estimates and also which features of regulatory regions might affect such estimates. We found that binding sites are usually aligned better than their surrounding sequences (figures 2B &3C) but are still misaligned starting at very short divergence distances (figure 3A). For instance, given the approximate divergence of *Drosophila pseudoobscura *from *Drosophila melanogaster*, at 1.79 substitutions per site [78], according to our results, only about 40% of truly conserved binding sites should even be overlapping in alignments. Unless the rate of binding site turnover is high enough such that the number of sites that have turned over is much larger than the 60% of truly conserved sites that have been misaligned, its unlikely that such a comparison would be useful for studying binding site evolution. If 40% binding site conservation, however, is higher than what might be expected in non-functional regions, then comparing these species might still be useful for predicting functional regulatory regions. Our finding that binding sites are often still overlapping in alignments even if they are not perfectly aligned (figure 3B) suggests that binding sites are not always strong alignment anchors, that small indels could lead to small alignment errors and that methods for identifying conserved binding sites that do not rely on perfect alignments would have greater sensitivity [21,28,79] (the specificity of such methods, however, would need to be explored to understand their predictive power). Finally we found that the higher the density of sites in an enhancer, the higher the alignment accuracy of the binding sites within, presumably due to the overall higher constraint and suppression of indels. Bacterial and yeast regulatory regions, for instance, are not understood to contain such high-density arrays of binding sites as metazoans [80,81] and would therefore be expected to align more poorly, all else being equal. Although we also found that longer binding and more highly specified sites are easier to align, this requires further investigation with a larger panel of transcription factors. The variance in alignment accuracy introduced by such regulatory sequence properties is important to consider before determining the expected error from simulations or before interpreting an evolutionary comparison across regulatory regions.

The second inference we considered was divergence distance estimation. We were impressed that our estimates using PAML's Baseml program on the true alignments generated in our simulations were highly accurate out to rather high divergences, suggesting that saturation does not lead to inaccuracies at short divergence distances, at least when the right model is used (figure 4A &4B). Because of the accuracy of the divergence inference step, we were able to look directly at the contribution of alignment error to divergence estimation. Although the tendency of two of the tools to overestimate divergences at short divergence distances is noteworthy (as was observed for Clustalw in [8]), most striking is the behavior that all tools reach a unique divergence distance at which divergence estimates cease to increase (figures 4A &4B) (this underestimate was also observed for Clustalw in [8]). This point of maximum divergence corresponded with the point at which tools reached their minimum alignment accuracy or where they were essentially randomly aligned (figure 4C). Shabalina and Kondrashov previously reported that unrelated sequences produce alignments that have a greater percent identity than would be theoretically predicted from base composition, suggesting that alignment tools add gaps to create more matches and fewer mismatches in order to maximize their scores [70]. The "twilight zone" (the point where alignments become random) [82] is therefore not 25% identity but instead is a much shorter divergence (or higher identity) which varies across alignment tools. For instance, pairwise alignments generated by Mavid reach the point where divergence estimates cease to increase at about 0.5 substitutions per site, which is approximately the divergence estimated for human and mouse, suggesting that fast evolving human or mouse sequences would on average not be detected as such from Mavid alignments. It is worth noting that Tba, stops returning alignments before it reaches the point where divergence estimates cease to increase, suggesting that the scoring scheme Tba uses to filter its alignments can avoid producing random alignments but also that it might fail to return an alignment with some remaining phylogenetic signal.

Our findings that overall alignment accuracy, binding site alignment accuracy and divergence estimation accuracy vary greatly across branches in a tree have profound implications for phylogenetic research of noncoding DNA. All four of the tools we examined exhibit systematic biases toward higher accuracy along branches connecting sister taxa relative to branches connecting internal nodes (figures 2E, 2F, 3D &4D). Consider the example of studying binding site turnover rates relative to substitution rates in human, mouse and rat alignments. Even if these rates were constant across the tree, binding site turnover might be detected as higher along the human branch because of increased alignment error along the longer node-to-leaf branch and substitution rates might be underestimated along the human branch because it is longer than an alignment tool's maximum divergence. Theses two biases combined would then cause turnover events per substitution to be even more distorted along the human branch. These results strongly suggest that either new alignment tools that minimize this bias or new phylogenetic methods that control for this bias need to be developed.

## Conclusion

Errors in the alignment of noncoding DNA are systematic phenomena that affect evolutionary inferences, decreasing accuracy and biasing results. In order to use the rich diversity of variation in more diverged sequences, new alignment and phylogenetic methods need to be developed to reduce and control for errors in automated alignment.

## Methods

### CisEvolver

CisEvolver was written in Perl. It is available for download [59].

### Trees

For both the divergence estimation and binding site conservation estimation simulations, each divergence distance tested was transformed into a Newick formatted tree. Figure 1 shows how divergences were distributed across trees.

### Divergence simulations

For the divergence estimation simulations, 100 simulations were run for each divergence distance. For each simulation, a 10 kb ancestral sequence was randomly generated from the *D. melangaster *mono-nulceotide base frequencies (60/40 AT/CG). The 10 kb sequences were evolved from the root node of the tree down the branches to leaves using a substitution and indel model. Substitutions occurred according to the HKY85 substitution model [54], using the *D. melanogaster *mono-nucleotide base frequencies and kappa set to 2.0 as has been observed in *Drosophila *[83]. Indel events occurred according to a Poisson indel event model:

*p*_*indel *_= 1 - *e*^-*Rk*^

where *R *is the relative rate of indels to substitutions and *k *is the length of the branch. In *Drosophila *indels have been found to occur approximately 10% the rate of substitutions so we used *R *= 0.1 [84,85]. Indel lengths were determined by a frequency distribution derived from *D. melanogaster *indel polymorphisms with a maximum of 58 bp [55]. Insertions and deletions were treated identically.

### Cis-regulatory sequences

Thirty-six experimentally characterized *cis*-regulatory regions that have been found to drive expression patterns in reporter assays recapitulating some or all of the expression pattern of an adjacent gene were collected from two recent papers on anterior/posterior patterning in *D. melanogaster *[26,60]. The sequences were mapped to release 4.0 of *D. melanogaster *using BLAT [86]. A GFF file with the enhancer coordinates is available in additional file 1: Enhancers.gff.

### Transcription factor binding sites

The 36 *cis*-regulatory regions used in the study have been reported to be bound or predicted to be bound by some combination of the following factors: Bicoid [61], Caudal [61], Giant [62], Hunchback [62], Knirps [62], Kruppel [62], Tailless [62] and Torso-response element [60]. Position weight matrices (PWMs) were either taken from published resources [60,61] or were generated from published footprints [62] using MEME [87] (described at [88]). Matrices are available in additional file 2: Matrices.txt.

For each of the 36 *cis*-regulatory regions, PASTER [89] was used to find sites with a p-value less than 10^-3 ^for each of the eight PWMs. If sites were overlapping one was randomly chosen and the others were thrown out.

### Transcription factor binding site conservation simulations

For the binding site conservation simulations, 25 replicates for each of the 36 *cis*-regulatory regions were evolved to each of the divergence distances. Sequences were evolved from the root down the branches of each tree using either a background or binding site mutation model. Non-binding site sequences in the enhancers were evolved according the HKY85 and indel models described above. Binding sites were evolved according to the HB98 substitution model [56]. We have previously shown that there is position-specific variation in substitution rates across functional binding sites and that the HB98 substitution model accurately predicts the relationship between the degeneracy of positions in a PWM and the position specific substitution rate across binding sites [28,57]. The rate of change from residue a to b at position *i *in the binding site is given by:

R(i)ab=Qablog⁡(fibQbafiaQab)1−fiaQabfibQba,
 MathType@MTEF@5@5@+=feaafiart1ev1aaatCvAUfKttLearuWrP9MDH5MBPbIqV92AaeXatLxBI9gBaebbnrfifHhDYfgasaacH8akY=wiFfYdH8Gipec8Eeeu0xXdbba9frFj0=OqFfea0dXdd9vqai=hGuQ8kuc9pgc9s8qqaq=dirpe0xb9q8qiLsFr0=vr0=vr0dc8meaabaqaciaacaGaaeqabaqabeGadaaakeaacqWGsbGucqGGOaakcqWGPbqAcqGGPaqkdaWgaaWcbaGaemyyaeMaemOyaigabeaakiabg2da9iabdgfarnaaBaaaleaacqWGHbqycqWGIbGyaeqaaOWaaSaaaeaacyGGSbaBcqGGVbWBcqGGNbWzdaqadiqaamaalaaabaGaemOzay2aaSbaaSqaaiabdMgaPjabdkgaIbqabaGccqWGrbqudaWgaaWcbaGaemOyaiMaemyyaegabeaaaOqaaiabdAgaMnaaBaaaleaacqWGPbqAcqWGHbqyaeqaaOGaemyuae1aaSbaaSqaaiabdggaHjabdkgaIbqabaaaaaGccaGLOaGaayzkaaaabaGaeGymaeJaeyOeI0YaaSaaaeaacqWGMbGzdaWgaaWcbaGaemyAaKMaemyyaegabeaakiabdgfarnaaBaaaleaacqWGHbqycqWGIbGyaeqaaaGcbaGaemOzay2aaSbaaSqaaiabdMgaPjabdkgaIbqabaGccqWGrbqudaWgaaWcbaGaemOyaiMaemyyaegabeaaaaaaaOGaeiilaWcaaa@61F5@

where *Q *is the background substitution model (HKY85) and *f *is the PWM for the factor. Indel events were not permitted in binding sites and deletions from background sequences were not allowed to extend into binding sites.

### Alignments

Alignments were performed using default parameter values for each of the following tools: Clustalw [63], Mavid v0.9 [64], Mlagan v1.2 [65] and Blastz/Tba [7,66,67].

### Alignment accuracy

Alignment accuracy was defined as

Acc=CTSUCSU,
 MathType@MTEF@5@5@+=feaafiart1ev1aaatCvAUfKttLearuWrP9MDH5MBPbIqV92AaeXatLxBI9gBaebbnrfifHhDYfgasaacH8akY=wiFfYdH8Gipec8Eeeu0xXdbba9frFj0=OqFfea0dXdd9vqai=hGuQ8kuc9pgc9s8qqaq=dirpe0xb9q8qiLsFr0=vr0=vr0dc8meaabaqaciaacaGaaeqabaqabeGadaaakeaacqWGbbqqcqWGJbWycqWGJbWycqGH9aqpdaWcaaqaaiabdoeadnaaBaaaleaacqWGubavcqWGtbWucqWGvbqvaeqaaaGcbaGaem4qam0aaSbaaSqaaiabdofatjabdwfavbqabaaaaOGaeiilaWcaaa@3ACA@

where *C*_*SU *_is the count of the ungapped columns in the simulated alignment and *C*_*TSU *_is the count of the ungapped columns in the simulated alignment that are aligned identically in the tool alignment. This measure is the same as "sensitivity" defined in [5].

Branch specific alignment accuracy was calculated similarly except that *C*_*SU *_was the count of ungapped columns for which the alignment was joining either sequences or correctly aligned sub-alignments and *C*_*TSU *_was the count of such columns in the simulated alignment that were aligned identically in the tool alignment. For instance, in a four species alignment, the node-to-node alignment accuracy was only based on the columns for which Seq1 and Seq2 were aligned correctly to each other and Seq3 and Seq4 were aligned correctly to each other (figure 1). Similarly, in a three species alignment, the node-to-leaf alignment accuracy was only based on the columns for which Seq1 and Seq2 were aligned correctly to each other. The motivation for this was to consider only the contribution to alignment accuracy a given branch contributes.

A script written in PERL that can calculate these measures is available for download [59].

### Binding site alignment measures

Binding site alignment was evaluated based on two measures. Sites that had the same start and stop position in each sequence in an alignment were considered to be perfectly aligned. Sites that were overlapping by at least one base in each of the sequence in an alignment were considered to be overlapping. The fraction of sites that were perfectly aligned and the fraction of sites overlapping in alignments across all *cis*-regulatory regions and all replicates are reported. The Pearson correlation between the density of binding sites in *cis*-regulatory regions and each measure as well as the correlation between the length of binding sites for each factor and each measure were calculated using the R statistics package[90].

### Divergence estimation

Divergence estimates were calculated using the baseml program from the PAML package v3.14 [69]. Baseml was run with the HKY85 model, estimating kappa with an initial value of 2, fixed alpha at infinity, no clock and estimating the equilibrium base frequencies from the observed averages.

## Authors' contributions

DAP designed the research, performed the research, analyzed the data and wrote the paper. AMM contributed to the development of the CisEvolver program. All authors contributed to the research design and the writing of the paper.

## Supplementary Material

Additional File 1 This file, in GFF2 format [91], contains the coordinates of the 36 enhancers used in this study in *Drosophila melanogaster *release 4 coordinates [92]. Click here for file

Additional File 2 This text file contains horizontal counts matrices and vertical frequency matrices for each of the eight transcription factors used in this study. Click here for file
